# Development of an assessment tool to measure communication skills among family medicine residents in the context of electronic medical record use

**DOI:** 10.1186/s12909-023-04216-1

**Published:** 2023-04-14

**Authors:** Jumana Antoun, Bassem Saab, Jinan Usta, Maya Romani, Imad Bou Akl, Maha Fathallah El Mofti, Joudy Eter, Natally AlArab, Hala Itani

**Affiliations:** 1 Department of Family Medicine, American University of Beirut, Lebanon, Lebanon; 2 Department of Internal Medicine, American University of Beirut, Lebanon, Lebanon

**Keywords:** Computer-based, Psychometrics, Assessment, Communication skills, Postgraduate

## Abstract

**Background:**

The introduction of the electronic medical record (EMR) has led to new communication skills that need to be taught and assessed. There is scarce literature on validated instruments measuring electronic-specific communication skills. The aim is to develop an assessment checklist that assesses the general and EMR-specific communication skills and evaluates their content validity and reliability.

**Methods:**

Using the SEGUE theoretical framework for communication skills, the assessment checklist items were developed by the Communication Skills Working Group (CSWG) at the family medicine department using a literature review about the positive and negative aspects of EMR use on physician-patient communication. A group of faculty members rated real resident-patient encounters on two occasions, three weeks apart. Patients were asked to fill out the Communication Assessment Tool (CAT) at the end of the encounter.

**Results:**

A total of 8 residents agreed to participate in the research, with 21 clinical encounters recorded. The average total score was 65.2 ± 6.9 and 48.1 ± 9.5 for the developed scale and the CAT scale, respectively. The scale reliability was good, with a Cronbach alpha of 0.694. The test-retest reliability was 0.873, p < 0.0001. For the total score on the developed checklist, the intraclass correlation coefficient between raters (ICC) was 0.429 [0.030,0.665], p-value of 0.019. The level of agreement between any two raters on the cumulative score of the 5 subsections ranged from 0.506 (interpersonal skills) to 0.969 (end encounter).

**Conclusion:**

This checklist is a reliable and valid instrument that combines basic and EMR-related communication skills.

**Supplementary Information:**

The online version contains supplementary material available at 10.1186/s12909-023-04216-1.

## Key messages

1- This is one of the few assessment tools developed to measure both basic and EMR-related communication skills.

2- The tool had good scale and test-retest reliability.

3- The level of agreement among a diverse group of raters was good.

**Supplementary Information:**

The online version contains supplementary material available at 10.1186/s12909-023-04216-1.

## Background

Electrical Medical Records (EMRs) have been widely implemented due to their proven ability to enhance care efficiency by increasing the time of physicians with their patients, limiting prescription errors, and promoting shared decision making [1]. On the other hand, the use of EMR has introduced a new set of EMR-specific communication skills because of its impact on eye contact, bridging trust, and the overall relationship between doctor and patient, and because of its effect on the overall room layout which may appear as an obstacle disabling proper communication [2]. This highlights the need to train physicians on how to balance the use of the computer and communication with the patients [3].

Many have proposed new skills, models, or curricula to integrate patient-centered communication within the medical visit in the era of EMR in the form of workshops [4], practice role-plays and brief didactics [5]. Nevertheless, there is a need for assessment tools for measuring physicians’ communication skills when using EMR. Many methods have been used to evaluate the residents’ communication skills in general, including (1) direct observation (Mini Clinical Evaluation Exercise (MINI-CEX) and video review); (2) standardized patients (Objective Standardized Clinical examinations- OSCE); (3) patient surveys; (4) self-assessment, and (5) peer evaluation (360-degrees evaluations). For direct observation, various validated checklists are employed to measure the communication skills of residents, namely “Kalamazoo Essential Elements: the communication checklist” [6], “MAAS – Global Rating List for Consultation skills of Doctors” [7], and the SEGUE Framework [8]. However, none of these checklists address the EMR-specific communication skills. A systematic review of the existing assessment tools for the evaluation of communication skills among physicians has found eight tools (out of 45 assessment tools) that were used more frequently but none of them tackled the EMR-specific communication skills [9]. Thus, there is a need to develop a validated tool that assesses EMR-specific communication skills among residents.

Only a few available articles tackled EMR-specific communication skills. Morrow et al., were the first to assess EMR communication skills using two checklists in pre-developed scenarios among first-year medical students after a brief educational intervention, one for basic communication skills while the other for EMR-related communication skills [5]. However, this study was conducted in 2007 when EMRs were just being used in the clinic and used a small sample size. By adopting the same checklist, Hassid et al. compared physicians’ scores on SEGUE and Morrow et al.’s EMR-specific communication skills checklist during videotaped simulated medical encounters [3]. There was a difference in the scores between both tools with consistent lower scores on EMR-specific communication skills. Similarly, Biagoili et al. assessed the EMR-specific communication skills of students while using EMR in a simulated environment, extending Morrow et al.’s work to include EMR-related data management skills while interacting with patients [10]. However, all three articles adopted checklists that were not formally validated. In 2017, the first validated tool was developed by Alkhureishia et al. as an Electronic-Clinical Evaluation Exercise tool (e-CEX) to test the EMR-communication skills of second-year medical students in the context of OSCE [11]. The checklist included 10 items related to EMR-specific communication skills only. Therefore, this study aims to develop and validate the psychometric properties of a single checklist that would include both the basic and EMR- related communication skills in the context of direct observation of real patients in a family medicine residency program.

## Methods

### Scale development

#### Items development

An extensive literature review was conducted, focusing on the use of computer and electronic medical records in the clinical setting, as well as the impact on communication skills. The findings of the literature review provided a good understanding of the doctor-patient-computer triad, which is influenced by both the physician’s clinical and interpersonal skills. To develop the measurable items for clinical skills, the SEGUE framework (setting up the stage, eliciting information, providing information, understanding patient perspective, concluding the interview) was selected. This framework has been noted to have a high level of acceptability, the ability to be used reliably, evidence of validity, and the ability to apply to a variety of contexts [12]. A set of 28 carefully selected items were developed and distributed across the framework’s various branches. The items were chosen based on the studies that found a positive or negative correlation with specific behaviors during the medical interview incorporating the computer. Items include interacting with the patient rather than the computer at the beginning of the interview [13, 14], avoiding the use of a computer when addressing a psychological burden [15], alternating gazes between screen and patient [16], and spatial rearrangements of the room for easy access to all members of the triad [17]. As a result, these items, among others, were used to create the final assessment tool. A set of skills representing relational and process-oriented items were included for the physician’s interpersonal skills. Examples include the physician’s ability to maintain an empathic approach while not being distracted by the computer’s presence, physician comfort during the interview process despite the presence of the computer, and, most importantly, the ability to maintain a patient-centered interview while incorporating the computer.

#### Scoring

A scaled grading approach was used to account for the presence and the quality of the measured skill or item. The scale for the clinical skills included a Likert scale, which is commonly used in medical education assessment and allows to reduce measurement sensitivity and differentiation in the quality of their performance on each specific task [18]. The Likert scale ranged from not done (0), poorly done, adequately done, and well done (6), with an option of “not applicable” included. The same approach was used in grading interpersonal skills, but the emphasis was on the physician maintaining the measurable attributes throughout the interview. The rater would be asked to rate the overall resident’s interpersonal skills during the encounter based on a Likert scale ranging from absent (0), not consistently applied, consistently applied, to exceptionally applied (6).

The total score was calculated as the sum of the scores on each item divided by the number of applicable items multiplied by 100. The same method of scoring is applied to the 5 subcategories of the checklist (setting the stage, eliciting information, giving information, understanding patient perspective, ending the encounter and interpersonal skills).

#### Content validity

A list of proposed items was developed. The communication skills working group (CSWG) at the family medicine department at the American University of Beirut includes four family physicians. The CSWG were considered the expert panel and every member was asked to rate every proposed item individually on a 5-Point-Likert scale that ranges from “totally agree” to “totally disagree.” They provided comments on proposed items, sentence structure and were free to suggest new items. An in-person meeting followed where all the collective results of their individual ratings were discussed. Each item, especially the ones that most of the groups disagreed upon, was discussed concerning the importance and clarity of the statement. A modified set of the items was developed and sent again for the CSWG members to rate them on their own. This process kept going till we reached the final set of items where at least 3 members agreed upon. Three rounds were performed, and the final set of items can be found in Appendix 1.

### Implementation of the scale

All the family medicine department residents’ clinics are equipped with a ceiling-mounted camera that captures part of the room where the history taking occurs. The examination table area is not captured. There is a sign in all the residents’ rooms stating the presence of the camera surveillance. The current practice in the clinic is that the preceptor can monitor any resident through the video monitor. The clinic’s policy mandates that the patient signs a written consent only if the encounter is videotaped; it is the nurse’s responsibility. Each resident has prescheduled clinic sessions per month. While the resident is attending to actual patients during a session, a faculty member sits in the preceptor room where the video monitor is present.

During the research period, the assessment nurse approached all the patients visiting the second- and third-year residents who agreed to participate in the research. The nurses requested permission from the patients to videotape the interview. If they agreed, the nurse obtained their signatures on the necessary forms, including the appropriate forms per the clinic policy and the research-related informed consent. The nurse then handed the patients a questionnaire, Communication Assessment Tool (CAT), which they were to fill out privately in the waiting area after their visit with the resident and asked to return the questionnaire in a sealed envelope. The relevant resident-patient encounter was retrieved from the surveillance system and saved in a password-protected folder. The same code was assigned to both the recorded video and the CAT.

The family medicine residency program is a three-year training program, accredited by the Accreditation Council for Graduate Medical Education- International (ACGME-I). Residents who plan to sit for the Arab board can have a four-year program. Training occurs in the main family medicine practice center at the American University of Beirut along with other satellite clinics. First-year residents were excluded because they have infrequent clinic sessions and are still learning how to use the electronic system. Last-fourth-year residents were also excluded as they spent most of their time in clinics located outside the main center.

### Psychometric properties of the assessment tool

Eight raters were assigned to rate the residents’ recorded encounters based on the developed scale. Every rater evaluated the same video encounter twice, three weeks apart. We aimed to have a diversified group of raters. The group of raters included the members of the CSWG, a faculty member who is the physician lead for assessments at the medical school and associate program director for the Internal Medicine residency program, two recently graduated medical doctors (to give their perspective as students), and the senior graduate medical education (GME) program coordinator at the department of family medicine (to give her perspective as patient with some experience in medical education). The raters completed an evaluation form about the ease of administering the assessment tool, including its friendliness and length.

The above procedure allows for measurement of test-retest reliability as the same rater evaluated the same video encounter on two occasions, separated by three weeks. Inter-rater reliability was measured by comparing the ratings of different preceptors of the same video on individual items and the overall score. The criterion validity of the checklist was measured by comparing the residents’ scores on the developed checklist to the patient’s CAT score. A variety of medical cases with varying chief complaints ensured generalizability.

### Statistical analysis

Descriptive analysis was performed to describe the number of residents, clinical encounters, scores on each item and total score, and the satisfaction of the raters. Cronbach’s alpha was used to measure the scale reliability. The interclass coefficient and Pearson correlation were used to measure inter-rater reliability and test-retest reliability, respectively. Each encounter was either rated by two or four raters, depending on the availability of the raters. For inter-reliability, all permutations of paired raters were used to calculate the interclass coefficient and one-way random-effect model was used. Spearman correlation was used to compare the developed scale score and CAT score as the CAT score was not normally distributed. P-value was set at 0.05 for statistical significance. SPSS version 27 was used for statistical analysis.

## Results

A total of 8 residents agreed to participate in the research. The study extended over one academic year. Twenty-one clinical encounters were recorded. The average length of the encounters was 15.6 ± 6.3 min. Most of the encounters were for acute complaints: foreign body in the eye, musculoskeletal complaints, chest pain, fever, diarrhea, urinary symptoms, upper respiratory tract infections, with very few included general chief complaints such as checkups, ordering some lab test and well-baby. The age of the patients varied between 5 and 62 years old, with 61.9% being female patients.

The average total score was 65.2 ± 6.9 and 48.1 ± 9.5 for the developed scale and the CAT scale, respectively. The scoring of each item is shown in Appendix 2. The correlation between the CAT score and the developed checklist score was 0.215, p-value 0.461. The scale reliability was good, with a Cronbach alpha of 0.694. The test-retest reliability was 0.873, p < 0.0001.

Some encounters were rated by more than 2 raters. The final analysis was based on a total of 52 pairs. For the total score on the developed checklist, the intraclass correlation coefficient between raters (ICC) was 0.429 [0.030,0.665], p-value of 0.019 (Table 1). The levels of agreement between any two raters for the individual items of the assessment criteria ranged from kappa = 0.359 (item 3) to kappa = 0.693 (item 4) (data not shown). The levels of agreement between any two raters on cumulative score of setting the stage was not significant. The level of agreement between any two raters on a cumulative score of the other 5 categories ranged from 0.506 (interpersonal skills) to 0.969 (end encounter). The level of agreement between any two raters on all the items was highest among the pair of family medicine/graduate medical student followed by the pair of two-family physicians (Table 2).

**Table 1:** Interclass reliability of total and category score paired ratings (N of cases = 52)

	Intraclass correlation coefficient [95% Confidence interval]	p-value
Total Score	0.429 [0.030, 0.665]	0.0190
Setting the stage	0.047 [-1.659,0.082]	0.3670
Eliciting information	0.578 [0.366, 0.734]	< 0.0001
Giving information	0.709 [0.544,0.822]	< 0.0001
Understand patient perspective	0.655 [0.468,0.786]	< 0.0001
End encounter	0.969 [0.924, 0.988]	< 0.0001
Interpersonal skills	0.506 [0.266, 0.687]	< 0.0001

**Table 2:** Interclass reliability of all the items scores by specialty of raters

	N of cases	Intraclass correlation coefficient [95% Confidence interval]	p-value
Internal medicine/family medicine	155	0.268 [0.01,0.460]	0.021
Family medicine/family medicine	237	0.349 [0.159, 0.496]	0.001
Family Medicine/administrator	183	-0.052 [-0.410,0.215]	0.633
Family medicine/student	613	0.564 [0.489,0.628]	< 0.001
Student/administrator	161	0.210 [-0.059,0.413]	0.058

Regarding the use of the assessment tool, all 7 raters totally agreed/agreed that the length of the assessment tool was adequate. One rater disagreed on the statement that it was easy to observe and evaluate the behavior. Two raters considered some of the sentences to be unclear or not easy to understand.

## Discussion

Appropriate use of EMR while still maintaining meaningful and engaging interaction with patients is an important skill. The literature is scarce regarding validated assessment tools to measure EMR related communication skills. This study aimed to develop and validate one single checklist that tackles both general communication skills and EMR-related communication skills of family medicine residents. The scale reliability was good, with a Cronbach alpha of 0.694. The test-retest reliability was 0.873, p < 0.0001. The level of agreement between any two raters on the total checklist score was 0.429 [0.030,0.665]. Although the interrater reliability was poor-moderate for the total scale score, the interrater reliability was moderate for eliciting information, giving information, understanding patient perspective and interpersonal skills and excellent in ending the encounter section. Setting the stage had the least interrater reliability of 0.047. Two items related to setting the stage were scored low by the raters, mainly introducing the computer, and reassuring the patient regarding confidentiality of EMR. With the expanded use of the computers in the daily activities, it is possible that physicians do not feel the need to introduce the computer. Patients consider the use of EMR in the clinic as a normal process and part of the physician’s work [19]. Moreover, physicians may consider that confidentiality of data is standard of care and does not need to be explained to the patient in every single encounter except in specific cases where sensitive information is going to be discussed.

The literature is scarce regarding checklists that measure EMR-related communication skills to compare the validity and reliability of the tool. The most relevant validated tool is the e-CEX developed by Alkureishi et al. among medical students [11]. In the e-CEX validation, the authors have studied discriminant validity between the e-CEX and standardized patients’ score and did not measure interrater reliability. In this study, we compared the checklist scores to the CAT score which is a reliable and valid instrument for measuring patients’ perception of physician communication skills in the context of EMR [20]. Nevertheless, there was a poor correlation between the CAT and checklist score. One explanation could be that patients tend to rate positively their physicians, or patients pay attention to different communication skills that academics look at. Another explanation is that CAT measures basic communication skills. Physicians who rated good on basic communication skills had lower scores on EMR related skills [3]. Further research should be conducted to measure the criterion validity by comparing to other faculty-based assessment measurements. Regarding the interrater reliability, the inter-class correlation coefficient of min-CEX clinical skills assessment among medical trainees ranged from 0.66 to 0.81 in different clinical scenarios [21]. A systematic review of 45 existing assessment tools to evaluate basic communication skills have shown poor-moderate psychometric properties [9]. Measuring communication skills is a challenging task given that it has a subjective component and may differ in different clinical settings such as medical students, specialty or practicing physicians.

Our study had several strengths like using a variety of rater backgrounds. Most of the literature on basic communication skills tools involves standardized patients in simulated environments where the learners are aware of their behaviors [9]. This study used videos of real patient encounters in a primary care setting. Moreover, this checklist combines both EMR and general communication skills. As for the study’s limitations, the residents involved in the study did not receive formal training in EMR-related communications skills. Moreover, the small number of residents who agreed to participate could lead to selection bias. Another limitation is the generalizability to other disciplines, especially that the interrater reliability between family medicine/internal medicine was low and it was based on a single institution.

### Practical and research implications

This tool is a valid starting point taking into consideration the lack of rigorous current checklists that measure EMR-related communication skills. This study has proven the validity and reliability of the tool. However, further research and optimization of the form is needed. It is worth re-structuring the form into three sections: basic skills, EMR-related skills and interpersonal skills. As EMRs become more established and standard of care in the future, some items may become obsolete that require a modified shorter form. A large sample with diverse types of residents may be warranted to increase generalizability of the tool. To improve the validity, this tool could be compared with other well established current basic communication skills tools. The scores of this tool could be compared to the overall scores of communicaiton skills captured by the program from other sources.

## Conclusion

This checklist is a reliable and valid instrument that combines both basic and EMR-related communication skills. Further research is needed to measure its psychometric properties in practice.

## Electronic supplementary material

Below is the link to the electronic supplementary material.

Supplementary Material 1

Supplementary Material 2

## Data Availability

All data generated or analyzed during this study are included in this published article.
