# Supplementary material for: Development of an assessment tool to measure communication skills among family medicine residents in the context of electronic medical record use
Source: BMC Med Educ. 2023 Apr 14;23:245. doi: 10.1186/s12909-023-04216-1 (PMC10103454; doi:10.1186/s12909-023-04216-1)
Supplement: Supplementary file 1 — Supplementary Material 1 [file 12909_2023_4216_MOESM1_ESM.docx]

**Appendix 1: The new proposed assessment tool to be filled by the preceptors after watching the videotaped encounter.**

Electronic Medical Record Related Communication Skills Evaluation Form

Instruction: Please rate the resident’s behaviors during the encounter based on the mentioned scale

| 0 | 1 | 2 | 3 | 4 | 5 | 6 | N/A |
| --- | --- | --- | --- | --- | --- | --- | --- |
| Not done | Poorly done | | Adequately done | | Well done | |  |

| Communication Skills |  |
| --- | --- |
| Setting the stage |  |
| Greeted patient appropriately and acknowledge waiting time if needed |  |
| Elicited chief complaint before turning to the computer |  |
| Introduced the computer into the Doctor – Patient – Computer triad |  |
| Explain and reassure patient of confidentiality of EMR |  |
| Rearranged spatial configuration so computer does not obstruct communication (screen in comfortable sharing position, physician not turning back to the patient…) |  |
| Eliciting Information |  |
| Established reason for visit |  |
| Established list of patient concerns and set an agenda for the encounter |  |
| Explored the patient’s psychosocial background |  |
| Intermittently looked at computer for previous relevant information while interviewing the patient |  |
| Pointed to relevant areas on the screen when needed |  |
| Looked at the patient and avoided computer use when patient is addressing a concern with a significant psychological burden |  |
| Involved patient in verifying EMR data entry’s accuracy and completion |  |
| Giving Information |  |
| When sharing the screen, physician verified the patient’s ability to visualize the contents optimally |  |
| Summarized history and did not use medical jargon |  |
| Discussed patient results with on screen visual aids (graphs, charts…) when applicable |  |
| Encouraged patient to ask questions and checked for patient understanding |  |
| Collaboratively agreed on care plan with patient |  |
| Kept balanced eye contact within the triad while sharing information |  |
| Understand Patient’s perspective |  |
| Recognized the patient’s perspective on the use of the computer in the clinic (such as typing while listening, the extent of computer use during the encounter, etc.) and acted accordingly |  |
| End Encounter: |  |
| Asked if patient would like to add anything |  |
| Shared patient education materials |  |
| Informed patient about follow up visit |  |

Please rate the overall resident’s skills during the encounter based on the mentioned scale

| 0 | 1 | 2 | 3 | 4 | 5 | 6 |
| --- | --- | --- | --- | --- | --- | --- |
| Absent | Not consistently applied | | Consistently applied | | Exceptional application of that skill | |

| Interpersonal Skills |  |
| --- | --- |
| Comfortable and proficient in using EMR and computer |  |
| Showed empathy |  |
| Maintained balanced eye contact between doctor and patient |  |
| Encouraged a partnership between doctor and patient |  |
| Overall conducted smooth organized interview and kept communication open and flowing |  |
