# Supplementary material for: Development of an assessment tool to measure communication skills among family medicine residents in the context of electronic medical record use
Source: BMC Med Educ. 2023 Apr 14;23:245. doi: 10.1186/s12909-023-04216-1 (PMC10103454; doi:10.1186/s12909-023-04216-1)
Supplement: Supplementary file 2 — Supplementary Material 2 [file 12909_2023_4216_MOESM2_ESM.docx]

Appendix 2: Mean score on each item (N=52 pairs)

|  | Communication skill | Rater 1 | Rater 2 |
| --- | --- | --- | --- |
| Item 1: Greeted patient appropriately and acknowledged waiting time if needed | Basic | 3.8±1.8 | 4.7±5.4 |
| Elicited chief complaint before turning to the computer | EMR-related | 3.8 ±1.6 | 4.2±1.4 |
| Introduced the computer into the Doctor – Patient – Computer triad | EMR-related | 0.37±1.2 | 0.46±1.5 |
| Explain and reassure patient of confidentiality of EMR | EMR-related | 0.87±1.9 | 1.1±2.4 |
| Item 5: Rearranged spatial configuration so computer does not obstruct communication (screen in comfortable sharing position, physician not turning back to the patient…) | EMR-related | 4.3±2.7 | 5.4±2.0 |
| Item 6: Established reason for visit | Basic | 3.8±1.2 | 4.3±1.3 |
| Item 7: Established list of patient concerns and set an agenda for the encounter | Basic | 2.6±1.7 | 3.8±1.9 |
| Item 8: Explored the patient’s psychosocial background | Basic | 2.6±1.9 | 2.6±2.1 |
| Item 9: Intermittently looked at computer for previous relevant information while interviewing the patient | EMR-related | 3.3±1.9 | 3.4±1.7 |
| Item 10: Pointed to relevant areas on the screen when needed | EMR-related | 3.6±3.1 | 5.2±2.2 |
| Item 11: Looked at the patient and avoided computer use when patient is addressing a concern with a significant psychological burden | EMR-related | 4.7±2.3 | 6.0±1.2 |
| Item 12: Involved patient in verifying EMR data entry’s accuracy and completion | EMR-related | 2.3±2.5 | 2.9±2.4 |
| Item 13: When sharing the screen, physician verified the patient’s ability to visualize the contents optimally | EMR-related | 4.6±2.9 | 5.3±2.7 |
| Item 14: Summarized history and did not use medical jargon | Basic | 3.2±1.9 | 2.0±1.8 |
| Discussed patient results with on screen visual aids (graphs, charts…) when applicable | EMR-related | 5.4±2.6 | 6.0±2.1 |
| Item 16: Encouraged patient to ask questions and checked for patient understanding | Basic | 3.3±1.6 | 2.9±1.6 |
| Item 17: Collaboratively agreed on care plan with patient | Basic | 3.5±1.1 | 3.5±1,.3 |
| Item 18: Kept balanced eye contact within the triad while sharing information | EMR-related | 4.3±2.0 | 4.5±1.6 |
| Item 19: Recognized the patient’s perspective on the use of the computer in the clinic (such as typing while listening, the extent of computer use during the encounter, etc.) and acted accordingly | EMR-related | 4.3±2.7 | 5.3±2.4 |
| Item 20: Asked if patient would like to add anything | Basic | 2.1±2.0 | 1.8±2.1 |
| Item 21: Shared patient education materials | Basic | 2.9±2.8 | 2.1±2.5 |
| Item 22: Informed patient about follow up visit |  | 3.0±2.4 | 4.1±2.7 |
| Item 23: Comfortable and proficient in using EMR and computer |  | 3.0±1.4 | 3.2±1.2 |
| Item 24: Showed empathy |  | 3.1±1.2 | 3.5±1.0 |
| Maintained balanced eye contact between doctor and patient |  | 3.8±1.3 | 4.1±1.1 |
| Item 26: Encouraged a partnership between doctor and patient |  | 3.5±1.1 | 3.6±0.9 |
| Overall conducted smooth organized interview and kept communication open and flowing |  | 3.6±0.8 | 4.0±0.7 |
